# The Influence of Education and Apolipoprotein *ε*4 on Mortality in Community-Dwelling Elderly Men and Women

**DOI:** 10.1155/2018/6037058

**Published:** 2018-03-25

**Authors:** Duke Appiah, Richard N. Baumgartner

**Affiliations:** ^1^Department of Public Health, Texas Tech University Health Sciences Center, 3601 4th Street, STOP 9403, Lubbock, TX 79430, USA; ^2^Department of Epidemiology and Population Health, School of Public Health and Information Sciences, University of Louisville, 485 E. Gray St., Louisville, KY 40202, USA

## Abstract

We investigated the risk of death in relation to the apolipoprotein *ε*4 allele and evaluated how it interacts with education in 504 elderly adults (mean age 73 years, 65.3% women) who were enrolled in 1993 into the New Mexico Aging Process Study. During 9 years of follow-up, apolipoprotein *ε*2 appeared to be associated with a lower risk for all-cause mortality (hazard ratio (HR) = 0.73, 95% confidence interval (CI): 0.30–1.71) compared to apolipoprotein *ε*3 carriers in models adjusted for age, sociodemographic variables, medical conditions, adiposity, and lifestyle factors. The apolipoprotein *ε*4 allele conferred almost a threefold elevated risk of mortality (HR = 2.76, CI: 1.42–5.37). An interaction between education and apolipoprotein e4 (*p*=0.027) was observed with the HR of mortality among e4 carriers compared to noncarriers being 1.59 (0.64–3.96) for those with ≥college education; 6.66 (1.90–23.4) for those with some college or trade; and 14.1 (3.03–65.6) for participants with ≤high school education. No significant interaction was identified between apolipoprotein E genotype and cognitive function for mortality risk. These findings suggest that genetic (apolipoprotein *ε*4) and environmental (education) factors act interactively to influences survival in the elderly with higher education attenuating the adverse effect of apolipoprotein *ε*4 on mortality.

## 1. Introduction

Apolipoprotein E (APOE), with regard to longevity, can be said to be the most extensively studied gene [1]. APOE, a 299 amino acid plasma protein with its gene found on chromosome 19, plays a central role in lipid transport and metabolism and also possesses several immunomodulatory properties such as suppressing lymphocytic T cell proliferation [2–4]. It has three polymorphisms: *ε*2, *ε*3, and *ε*4 with the later being associated with increased susceptibility for several chronic diseases as well as mortality [5–7], while *ε*2 has been suggested to have a protective effect, compared to *ε*3 [8].

Reports on the association of APOE allelic variation with mortality has been equivocal with differences in estimates observed among different ethnic/racial populations [9–13]. Some of the possible explanations for the inconsistent results across studies is that APOE *ε*4 allele is associated with Alzheimer's and cardiovascular disease, two conditions that in themselves are among the most prevalent causes of mortality in the elderly, resulting in survival bias with reduced frequency of the e4 allele in persons aged 65 and older [8]. Additionally, secular trends and geographic variation in the rates of the aforementioned chronic diseases suggest that the risk of death conferred by APOE genotype may depend on complex interactions with various environmental or behavioral risk factors over the lifespan of an individual. In this regard, numerous reviews have raised the issue that most studies assessing the effect of APOE across populations and time ignore the effects of behavioral factors that predisposes people to disease [1].

A large body of research has demonstrated a positive association of educational attainment with better health outcomes [14, 15] as well as reduced mortality [16]. Accordingly, over the last decades, reports from numerous studies allude to a gene-environment interaction between APOE genotype and education on health outcomes, namely, dementia [17], brain structure and development [18, 19], and cognitive function [20–22]. However, observations concerning the possible modifying effect of educational status on mortality in the elderly, independent of chronic comorbidities, are limited. Therefore, we aimed to evaluate whether educational status would offset the adverse influence of the APOE *ε*4 allele on mortality among community-dwelling elderly adults.

## 2. Materials and Methods

The New Mexico Aging Process Study (NMAPS) was a longitudinal study conducted between 1979 and 2001 in Albuquerque, NM, to describe the nutritional status and factors associated with healthy aging in community-dwelling elderly persons. The study design, subject recruitment, and inclusion and exclusion criteria have been described in detail previously [23]. Briefly, to be enrolled in the study, a person had to be 60 years and older and have no preexisting medical conditions such as cancer (with the exception of nonmelanoma skin cancer), recent myocardial infarction, type 2 diabetes mellitus, chronic obstructive pulmonary disease, or uncontrolled hypertension. About 90% of participants were non-Hispanic whites, 8% of Hispanic origin, and close to 2% being nonwhite. An annual visit was required for all participants in whom blood samples were obtained, and body composition, physical, functional, cognitive, and nutritional status were assessed. The yearly dropout rate for the study was approximately 3.6% with major chronic conditions and migration out of Albuquerque being the main reasons. The study was approved by the Human Subjects Research Review Committee of the University of the New Mexico School of Medicine with all participants providing informed consent in accordance with the Declaration of Helsinki, and all methods were carried out in accordance with the relevant guidelines and regulations. Participants who were alive at 1993, were followed up for 9 years (median, 7 years), and had genotype data together with measures of cognitive function were included in the current analysis.

APOE genotypes were assessed by restriction fragment length polymorphism analysis of polymerase chain reaction products, as previously developed [24, 25]. The assays were conducted in the Aging and Genetic Epidemiology (AGE) laboratory [23]. Of the 577 individuals who participated at baseline, 519 had APOE genotyped successfully. To distinguish unambiguously the effect of the APOE allele and genotype, we further excluded participants with APOE 2/4 (*n*=15), resulting in an analytic sample of 504 participants.

Cognitive function was evaluated by using the modified version of the Mini-Mental State Examination (3MS) [26]. Depression was measured using the short form of the Geriatric Depression Scale [27], with a score of 5 or higher suggestive of depressive symptomatology.

Educational status was assessed using a questionnaire with possible responses being less than 12 years of education (high school), high school graduate or passed the tests of general educational development, some college or technical school, college degree, some grad school, masters degree, and doctorate. Other covariates of interest were sex, marital status, body mass index (BMI), bone mineral density, smoking status, diastolic blood pressure, and levels of total cholesterol and triglyceride. BMI was calculated by dividing weight in kilograms by the square of height in meters. Bone mineral density was measured by Lunar DPX DXA (GE/Lunar Radiation Corp., Madison, WI). Chronic conditions including cancer, nonvertebral or vertebral fracture, diabetes, ischemic heart disease, and cerebrovascular disease were all self-reported at each annual visit, verified against medical records, and coded according to the International Classification of Diseases, Ninth Revision (ICD-9) codes. ICD-9 codes used were 140–208 for cancer; 250 for diabetes (or fasting blood glucose level ≥ 7 mmol/L); 290, 294, and 331 for dementia; 430–438 for cerebrovascular disease; 410–414 and 428–429 for ischemic heart disease; and 800–829 nonvertebral or vertebral fracture. Deaths were ascertained using the National Death Index and verified whenever possible against state death certificates and local obituary reports.

### 2.1. Statistical Analysis

Differences in baseline characteristics by APOE genotype were assessed using chi-square for categorical variables and ANOVA for continuous variables that were normally distributed or the Kruskal–Wallis test for nonnormally distributed variables. In time-to-event analysis, the Kaplan–Meier method was used to evaluate the incidence of all-cause mortality in relation to APOE genotype groups with the censoring date being December 31, 2002. Attained age was used as the time scale for all analyses as this procedure offers the opportunity to account for the effect of survival bias (thus individual with the highest risk of mortality are not observed) [28]. Furthermore, the use of attained age as the time scale provides a more straight-forward interpretation of mortality since it is free of the confounding effect of age which is intrinsically taken into account as a measure of survival time [29]. Survival curves were then produced to graphically depict incident events with differences among groups assessed by means of the log-rank test. Adjusted hazard ratios (HRs) and 95% confidence intervals (CIs) for mortality were calculated using Cox proportional hazards regression. Four models with progressive adjustments were used. Model 1 was adjusted for demographic variables such as age, sex, education, and marital status. Model 2 comprised model 1 with further adjustment for levels of triglyceride, BMI, bone mineral density, and diastolic blood pressure. Model 3 further adjusted for psychological factors such as scores on both the Geriatric Depression Scale and the Modified Mini-Mental State Examination. Finally, model 4 also adjusted for incident medical conditions, including all cancers, nonvertebral or vertebral fractures, diabetes, ischemic heart disease, and cerebrovascular disease. APOE status was identified with dummy coded indicator variables with the 3/3 genotype used as the referent. Interactions of APOE *ε*4 allele and educational as well as other statistically significant covariates on the risk of mortality were explored using the likelihood ratio test to compare models with and without the interaction terms. The assumption of proportional hazards was assessed by graphical methods using Schoenfeld residuals. To assess the robustness of the estimates, we performed a sensitivity analysis which included information for the 15 participants with APOE *ε*2/4. A two-tailed probability value less than 0.05 was considered statistically significant, and all analyses were performed using SAS software, version 9.4 (SAS Institute, Inc., Cary, NC)

## 3. Results

The mean age of participants at baseline was 73.4 (range 61–91) with 65.3% being women. Approximately half of the participants reported having a college degree or higher and only 11.3% had a BMI of 30 or higher. Nearly 5% of the cohort was identified as having depressive symptomatology indicative of depression, and less than 2% of participants were classified as having dementia. Several differences in baseline characteristics were observed between men and women. Men were more likely than women to be older and have higher bone mineral density and higher body weight, with a significantly greater proportion reporting having graduated from college (66.3% versus 47.1%), currently married (86.2% versus 44.9%), diagnosed with ischemic heart disease (14.3% versus 4.6%), and cancer (16% versus 10%). Women were more likely to have higher levels of total cholesterol and triglyceride with a significantly greater proportion reporting fractures (15.5% versus 8%). A description of the cohort according to APOE genotype is shown in Table 1. The most common genotype was APOE 3/3 found in 66.7% of participants with 13.9% and 18.1% having *ε*2/3 and *ε*3/4 genotypes, respectively. Additionally, only 19.2% of participants had at least one *ε*4 allele. These percentages are consistent with other prospective population studies among the elderly, especially those 60 years and older. As expected, total cholesterol levels varied across APOE genotype groups (*p*_for trend_ = 0.01) with APOE *ε*2 carriers having the lowest levels of total cholesterol.

**Figure 1:**
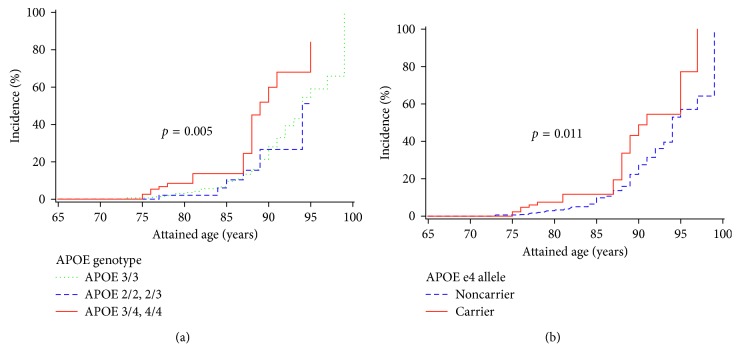
Kaplan–Meier cumulative incidence estimates for all-cause mortality by (a) APOE genotype and (b) APOE *ε*4 allele.

During follow-up, 61 (12%) deaths were recorded with fatal events occurring twice as often in men than women.  Figure 1 shows unadjusted Kaplan–Meier cumulative incidence estimates for all-cause mortality according to APOE genotype and allele. The log-rank statistic suggests that APOE genotype and allele influenced mortality, with APOE *ε*4 carriers having a significantly higher number of deaths. In multivariable models (Table 2), participants with the APOE *ε*4/*ε*4 genotype had a greater risk of mortality (HR = 2.56, 95% CI: 1.33–4.94) compared to persons homozygous for *ε*3 allele (model 3). APOE *ε*4 carriers had a twofold elevated risk of mortality compared to non-APOE *ε*4 carriers (HR = 2.21, 95% CI: 1.20–4.09). These estimates increased in magnitude after further adjustment for chronic medical conditions yielding hazard ratios of 2.61(CI: 1.33–5.14) and 2.76 (CI: 1.42–5.37) for APOE *ε*4/*ε*4 genotype and e4 allele carriers compared to persons homozygous for *ε*3 allele and non-APOE *ε*4 carriers, respectively.

Educational status appeared to be negatively associated with mortality with persons who had college education or more having lower risk of mortality compared to participants with high school education or less (HR = 0.79, 0.35–1.76). Howbeit, this did not approach statistical significance. Finally, we observed a significant interaction between educational status and APOE *ε*4 allele on the risk of mortality (*p*=0.027). The HR of mortality among *ε*4 carriers compared to noncarriers was 1.59 (0.64–3.96) for those with ≥college education; 6.66 (1.90–23.4) for those with some college or trade; and 14.1 (3.03–65.6) for participants with ≤high school education. No significant interaction was identified between APOE genotype and cognitive function for mortality risk. These results remained unchanged when information from the 15 participants with APOE *ε*2/4 was included in the analysis, and APOE *ε*2/4 was not significantly associated with mortality which could possibly be attributed to the small sample size (data not shown).

## 4. Discussion

In this community-based sample of elderly adults, we observed that the APOE *ε*3/4 and *ε*4/4 genotypes were associated with higher incidence of all-cause mortality, independent of important confounders. However, this elevated mortality risk was offset by higher educational status. To our knowledge, this is the first study to report that APOE (genetic factor) and education (environmental factor) act interactively to influences survival in the elderly.

While the underlying mechanism by which education ameliorates the influence of APOE genotype on mortality among elderly adults is a topic of major interest, it is not fully understood. It is possible that education may exert some beneficial effects on survival through two main avenues. That is, either through increased socioeconomic resources over the life course that could aid in the prevention of chronic diseases or through neuroprotective mechanism involved in reducing age-related or pathological changes in the structure and function of the brain which in the long term might influence longevity [30, 31]. On the one hand, higher education, more often than not, provides numerous resources such as higher income, stable employment, better social networks, health-related self-efficacy, and access to safe neighborhoods [32]. Accordingly, a large body of evidence has shown that higher educational attainment, whether measured as years of education or degree completion, is associated with reduced incidence of chronic morbidities [33], delayed mortality [34], and improved overall health primarily due to better access and utilization of health care and the adaption of health-promoting behaviors and lifestyles [14].

On the other hand, education may moderate the expression of the APOE *ε*4 allele on the risk of mortality in the elderly by influencing age-related or pathological changes in brain-matter and cognitive reserve. Some [35], but not all [36, 37], neuroimaging studies of cognitively healthy elders have found higher education to be associated with low accumulation of amyloid-beta deposition [38]; physiologically increasing regional cortical thickness [39]; increasing white and gray matter volume [35] in the superior temporal gyrus, insula, and anterior cingulate cortex [18]; decreasing mean diffusivity in the bilateral hippocampus [40]; and influencing a greater metabolism in the anterior cingulate cortex [18]. Furthermore, higher education is reported to be positively related with anterior cingulate connectivity which in turn is associated with improved cognitive performances [18]. These results and several others provide profound evidence that brain and cognitive reserve delays the clinical manifestations of dementia [18]. Compared to persons without the APOE *ε*4 variant, those with two copies of the *ε*4 variant are known to have more than a sevenfold elevated risk of developing Alzheimer's disease [38], the 6th leading cause of mortality in the United States. Taken together, these results and that of the present study suggest that higher education may offset the negative effects of the APOE *ε*4 on mortality by increasing cerebral reserve capacity against Alzheimer's disease and other late-life cognitive-related diseases. Even among elderly adults with Alzheimer's disease, a history of higher education has been reported to aid in coping better with the effects of brain atrophy by increasing cognitive reserve [39].

There is a strong, positive, and well-documented relation between APOE genotype and morbidity and mortality, and our results are consistent with some [12, 41–46] but not other studies [2, 8–11, 13, 47, 48] conducted among the elderly. For instance, the Cache County study with 4701 participants aged 65 and older found a greater mortality risk in persons carrying the *ε*3/4 and *ε*4/4 genotype which persisted even after accounting for the effect of Alzheimer's and cardiovascular disease [42]. One of the several reasons for the diverging results for APOE and mortality may pertain to small samples. The improvement in statistical power offered by studies with longer follow-up, such as the NMAPS, is important because there is a greater chance of identifying an effect if it truly exists. The Rotterdam study which followed 6852 participants for a mean of 5.4 years [47] did not identify any association between APOE genotype and longevity. However, after follow-up was extended to a mean of 11.1 years [4], with improved risk factor adjustments, APOE *ε*2 carriers with normal weight were found to have a reduced risk of mortality. Other reasons for the divergent results could be due to the heterogeneity in the relative frequency of the APOE *ε*4 allele among ethnic/racial groups, as well as methodological issues including selection bias and inadequate adjustment for confounders, which can all potentially influence study results [49].

This study has several potential limitations. First, it was restricted to a sample of elderly community-dwelling persons who were predominantly Caucasians and generally in good health. Thus, the results may not be generalizable to other ethnic/racial groups or elderly community-dwelling persons. Second, not all participants enrolled in the study consented to give blood for genotyping. Although such participants were older, there was no difference with respect to mortality and chronic medical conditions between those who did and did not have genotype data. In any case, the effect of any difference on our results cannot be estimated. Because the distribution of APOE genotype was similar to that reported in other prospective cohort studies of elderly persons, we believe that our results are not seriously biased. Third, the sample size of the cohort was relatively smaller, and with the occurrence of fatal events not being common, the statistical power to detect very weak associations is limited. Nonetheless, the associations detected were strong and statistically significant. Finally, we did not observe any effect modification of chronic ailments such as cancer, impaired cognitive function, diabetes, ischemic heart disease, and cerebrovascular disease with APOE genotype on longevity. This may primarily be due to the low prevalence of these conditions in our cohort. Serious chronic medical conditions were the main reason for dropout from the NMAPS, followed by migration. However, persons who dropped out still contributed information to the analysis since they were censored at the last date known to be alive. Also, follow-up for mortality status in the cohort was nearly complete. Nonetheless, it remains possible that these medical conditions may play a moderating role in the relationship between APOE and mortality.

In summary, we observed that APOE *ε*4 allele is positively related to mortality risk with this elevated mortality offset by higher educational status; however, the mechanism underlying this association is not fully understood. Discovering candidate genes that influence longevity has great public health importance. With almost 15% of the general population known to have the APOE *ε*4 variant and this proportion increasing to about 40% among persons with Alzheimer's disease [38], the moderating effects of education, lifestyle, and behavioral factors on genes should be studied to achieve a better understanding of their influence on longevity. This will go a long way to aid in targeting interventions towards high-risk individuals which will in turn reduce morbidity and improve longevity as well as quality of life among such persons.

## Figures and Tables

**Table 1:** Baseline characteristics of participants in the New Mexico Aging Process Study.

Characteristics	Apolipoprotein E genotype group			*p* value
	APOE 2 carriers (*N*=71)	APOE 3/3 (*N*=336)	APOE 4 carriers (*N*=97)	
Age, years	73.8 (6.4)	73.5 (6.5)	72.4 (5.8)	0.261
Sex, %				0.110
Male	45.1	32.1	36.1	
Female	54.9	67.9	63.9	
Education, %				0.642
High school or less	16.9	17.2	15.5	
Some college or trade	36.6	28.3	27.8	
College degree or higher	46.9	54.5	56.7	
Marital status, %				0.160
Single	30.4	42.9	40.4	
Married	69.6	57.1	59.6	
Current smoker, %	7.3	3.9	3.2	0.361
Depressive symptoms, %	4.2	4.5	4.1	0.988
Diastolic blood pressure (mmHg)	80.4 (10.2)	79.5 (11.3)	79.5 (10.8)	0.805
Total cholesterol (mmol/l)	5.10 (0.75)	5.47 (0.93)	5.49 (1.04)	0.008
Triglycerides (mmol/l)	1.61 (0.82)	1.62 (0.99)	1.55 (0.86)	0.820
Body mass index (kg/m^2^)	25.4 (3.49)	25.5 (4.01)	24.7 (3.91)	0.252
Waist to hip ratio	0.92 (0.11)	0.88 (0.1)	0.89 (0.11)	0.062
Bone mineral density	1.10 (0.13)	1.07 (0.12)	1.09 (0.11)	0.145
Mini-Mental State Exam scores	94.6 (4.91)	95.2 (4.28)	94.1 (5.73)	0.107
Medical conditions at follow-up				
Diabetes	4.2	3.3	4.1	0.817
Fractures	12.7	13.4	11.3	0.867
Cancers	5.6	12.5	15.5	0.144
Ischemic heart disease	7.0	7.4	10.3	0.626
Cerebrovascular disease	5.6	4.5	9.3	0.190
Deceased at the end of study	9.9	11.0	17.5	0.183

Values are mean (standard deviation) for continuous variables and percentages for categorical variables; *p* values based on the chi-square test for categorical variables and ANOVA for normally distributed continuous variables or the Kruskal–Wallis test for nonnormally distributed continuous variables.

**Table 2:** APOE genotype and allele as predictors of all-cause mortality.

	Unadjusted	Model 1	Model 2	Model 3	Model 4
Apolipoprotein E genotype					
APOE *ε*3/3	1	1	1	1	1
APOE *ε*2/2, 2/3	0.80 (0.34–1.80)	0.73 (0.32–1.65)	0.73 (0.32–1.68)	0.75 (0.32–1.74)	0.73 (0.31–1.71)
APOE *ε*3/4, 4/4	2.38 (1.33–4.26)	2.34 (1.30–4.36)	2.53 (1.35–4.73)	2.56 (1.33–4.94)	2.61 (1.33–5.14)
Apolipoprotein E allele					
APOE *ε*4 noncarrier	1	1	1	1	1
APOE *ε*4 carrier	1.99 (1.17–3.38)	2.20 (1.25–3.86)	2.26 (1.24–4.13)	2.21 (1.20–4.09)	2.76 (1.42–5.37)

Model 1: adjusted for age, sex, education, and marital status; model 2: model 1 plus levels of triglyceride, body mass index, bone mineral density, and diastolic blood pressure; model 3: model 2 plus Geriatric Depression Scale and the Modified Mini-Mental State Examination scores; model 4: model 3 plus incident medical conditions occurring during follow-up (cancers, nonvertebral or vertebral fractures, diabetes, ischemic heart disease, and cerebrovascular disease).

## References

[1] Lewis S. J., Brunner E. J. (2004). Methodological problems in genetic association studies of longevity–the apolipoprotein E gene as an example. *International Journal of Epidemiology*.

[2] Rockwood K., Nassar B., Mitnitski A. (2008). Apolipoprotein E-polymorphism, frailty and mortality in older adults. *Journal of Cellular and Molecular Medicine*.

[3] Zhang H. L., Wu J., Zhu J. (2010). The role of apolipoprotein E in Guillain-Barre syndrome and experimental autoimmune neuritis. *Journal of Biomedicine and Biotechnology*.

[4] Pardo Silva M. C., Janssens A. C. J. W., Hofman A. (2008). Apolipoprotein E gene is related to mortality only in normal weight individuals: the Rotterdam Study. *European Journal of Epidemiology*.

[5] Eichner J. E. (2002). Apolipoprotein E polymorphism and cardiovascular disease: a HuGE review. *American Journal of Epidemiology*.

[6] Farrer L. A. (1997). Effects of age, sex, and ethnicity on the association between apolipoprotein E genotype and Alzheimer disease. A meta-analysis. APOE and Alzheimer Disease Meta Analysis Consortium. *JAMA*.

[7] Lahoz C., Schaefer E. J., Cupples L. A. (2001). Apolipoprotein E genotype and cardiovascular disease in the Framingham Heart Study. *Atherosclerosis*.

[8] Fillenbaum G. G., Blazer D. G., Burchett B. M., Saunders A. M., Taylor D. H. (2002). Apolipoprotein E ε4 and risk of mortality in African American and white older community residents. *Gerontologist*.

[9] Lee J. H., Tang M.-X., Schupf N. (2001). Mortality and apolipoprotein E in Hispanic, African-American, and Caucasian elders. *American Journal of Medical Genetics*.

[10] Fillenbaum G. G., Burchett B. M., Lee J. H., Blazer D. G. (2003). Mortality and apolipoprotein E in African-American, and White elders: an attempted replication. *American Journal of Medical Genetics*.

[11] Lane K. A., Gao S., Hui S. L. (2003). Apolipoprotein E and mortality in African-Americans and Yoruba. *Journal of Alzheimer’s Disease*.

[12] Ewbank D. C. (2007). Differences in the association between apolipoprotein E genotype and mortality across populations. *Journals of Gerontology Series A: Biological Sciences and Medical Sciences*.

[13] Lima-Costa M. F., Peixoto S. V., Taufer M., Moriguchi E. H. (2008). Apolipoprotein e genotype does not predict 9-year all-cause mortality in brazilian older adults: the Bambui Cohort Study. *Journal of the American Geriatrics Society*.

[14] Boardman J. D., Domingue B. W., Daw J. (2015). What can genes tell us about the relationship between education and health?. *Social Science & Medicine*.

[15] Ross C. E., Mirowsky J. (1999). Refining the association between education and health: the effects of quantity, credential, and selectivity. *Demography*.

[16] Hummer R. A., Hernandez E. M. (2013). The effect of educational attainment on adult mortality in the United States. *Population Bulletin*.

[17] Wang H. X., Gustafson D. R., Kivipelto M. (2012). Education halves the risk of dementia due to apolipoprotein ε4 allele: a collaborative study from the Swedish brain power initiative. *Neurobiology of Aging*.

[18] Arenaza-Urquijo E. M., Landeau B., La Joie R. (2013). Relationships between years of education and gray matter volume, metabolism and functional connectivity in healthy elders. *Neuroimage*.

[19] Arenaza-Urquijo E. M., Gonneaud J., Fouquet M. (2015). Interaction between years of education and APOE epsilon4 status on frontal and temporal metabolism. *Neurology*.

[20] Van Gerven P. W., Van Boxtel M. P. J., Ausems E. E. B., Bekers O., Jolles J. (2012). Do apolipoprotein E genotype and educational attainment predict the rate of cognitive decline in normal aging? A 12-year follow-up of the Maastricht Aging Study. *Neuropsychology*.

[21] Strandberg T. E., Pitkala K., Eerola J., Tilvis R., Tienari P. J. (2005). Interaction of herpesviridae, APOE gene, and education in cognitive impairment. *Neurobiology of Aging*.

[22] Ishioka Y. L., Gondo Y., Fuku N. (2016). Effects of the APOE ε4 allele and education on cognitive function in Japanese centenarians. *Age*.

[23] Wayne S. J., Vellas B. J., Brodie S. G., Garry P. J., Baumgartner R. N. (2005). Apolipoprotein ε4 allele and problems with orientation are associated with a persistent decline in cognition in community-dwelling elderly persons. *Journals of Gerontology Series A: Biological Sciences and Medical Sciences*.

[24] Hixson J. E., Vernier D. T. (1990). Restriction isotyping of human apolipoprotein E by gene amplification and cleavage with HhaI. *Journal of Lipid Research*.

[25] Garry P. J., Wayne S. J., Vellas B. (2007). The New Mexico aging process study (1979–2003). A longitudinal study of nutrition, health and aging. *Journal of Nutrition, Health and Aging*.

[26] Teng E. L., Chui H. C. (1987). The Modified Mini-Mental State (3MS) examination. *Journal of Clinical Psychiatry*.

[27] Sheikh J. L., Yesavage J. A. (1986). Geriatric Depression Scale (GDS). Recent evidence and development of a shorter version. *Clinical Gerontologist*.

[28] Cain K. C., Harlow S. D., Little R. J. (2011). Bias due to left truncation and left censoring in longitudinal studies of developmental and disease processes. *American Journal of Epidemiology*.

[29] Lamarca R., Alonso J., Gómez G., Muñoz A. (1998). Left-truncated data with age as time scale: an alternative for survival analysis in the elderly population. *Journals of Gerontology Series A: Biological Sciences and Medical Sciences*.

[30] Cook C. J., Fletcher J. M. (2015). Can education rescue genetic liability for cognitive decline?. *Social Science & Medicine*.

[31] Bartres-Faz D., Arenaza-Urquijo E. M. (2011). Structural and functional imaging correlates of cognitive and brain reserve hypotheses in healthy and pathological aging. *Brain Topography*.

[32] Montez J. K., Berkman L. F. (2014). Trends in the educational gradient of mortality among US adults aged 45 to 84 years: bringing regional context into the explanation. *American Journal of Public Health*.

[33] Choi A. I., Weekley C. C., Chen S.-C. (2011). Association of educational attainment with chronic disease and mortality: the Kidney Early Evaluation Program (KEEP). *American Journal of Kidney Diseases*.

[34] Mackenbach J. P., Kunst A. E., Groenhof F. (1999). Socioeconomic inequalities in mortality among women and among men: an international study. *American Journal of Public Health*.

[35] Foubert-Samier A., Catheline G., Amieva H. (2012). Education, occupation, leisure activities, and brain reserve: a population-based study. *Neurobiology of Aging*.

[36] Christensen H., Batterham P. J., Mackinnon A. J. (2009). Education, atrophy, and cognitive change in an epidemiological sample in early old age. *American Journal of Geriatric Psychiatry*.

[37] Bastin C., Yakushev I., Bahri M. A. (2012). Cognitive reserve impacts on inter-individual variability in resting-state cerebral metabolism in normal aging. *Neuroimage*.

[38] Corder E. H., Saunders A., Strittmatter W. (1993). Gene dose of apolipoprotein E type 4 allele and the risk of Alzheimer’s disease in late onset families. *Science*.

[39] Liu Y., AddNeuroMed Consortium, Julkunen V. (2012). Education increases reserve against Alzheimer’s disease–evidence from structural MRI analysis. *Neuroradiology*.

[40] Piras F., Cherubini A., Caltagirone C., Spalletta G. (2011). Education mediates microstructural changes in bilateral hippocampus. *Human Brain Mapping*.

[41] Ewbank D. C. (2002). Mortality differences by APOE genotype estimated from demographic synthesis. *Genetic Epidemiology*.

[42] Hayden K. M., Zandi P. P., Lyketsos C. G. (2005). Apolipoprotein E genotype and mortality: findings from the Cache County Study. *Journal of the American Geriatrics Society*.

[43] Little D. M., Crooks V. C., Petitti D. B. (2009). Mortality, dementia, and apolipoprotein E genotype in elderly white women in the United States. *Journal of the American Geriatrics Society*.

[44] Corder E. H., Basun H., Lannfelt L. (1996). Attenuation of apolipoprotein E ε4 allele gene dose in late age. *The Lancet*.

[45] Corder E. H., Lannfelt L., Viitanen M. (1996). Apolipoprotein E genotype determines survival in the oldest old (85 years or older) who have good cognition. *Archives of Neurology*.

[46] Heijmans B. T., Slagboom P. E., Gussekloo J. (2002). Association of APOE ε2/ε3/ε4 and promoter gene variants with dementia but not cardiovascular mortality in old age. *American Journal of Medical Genetics*.

[47] Slooter A. J., Cruts M., Van Broeckhoven C., Hofman A., Van Duijin C. M. (2001). Apolipoprotein E and longevity: the Rotterdam Study. *Journal of the American Geriatrics Society*.

[48] Vogt M. T., Cauley J. A., Kuller L. H. (1997). Apolipoprotein E phenotype, arterial disease, and mortality among older women: the study of osteoporotic fractures. *Genetic Epidemiology*.

[49] Eto M., Watanabe K., Ishii K. (1986). A racial difference in apolipoprotein E allele frequencies between the Japanese and Caucasian populations. *Clinical Genetics*.

